# Evaluation of ^68^Ga-PSMA-11 PET-MRI in Patients with Advanced Prostate Cancer Receiving ^177^Lu-PSMA-617 Therapy: A Radiomics Analysis

**DOI:** 10.3390/cancers13153849

**Published:** 2021-07-30

**Authors:** Wolfgang Roll, Philipp Schindler, Max Masthoff, Robert Seifert, Katrin Schlack, Martin Bögemann, Lars Stegger, Matthias Weckesser, Kambiz Rahbar

**Affiliations:** 1 Department of Nuclear Medicine, University Hospital Muenster, 48149 Muenster, Germany; Robert.Seifert@ukmuenster.de (R.S.); Lars.Stegger@ukmuenster.de (L.S.); matthias.weckesser@ukmuenster.de (M.W.); Kambiz.Rahbar@ukmuenster.de (K.R.); 2 Department of Radiology, University Hospital Muenster, 48149 Muenster, Germany; philipp.schindler@ukmuenster.de (P.S.); max.masthoff@ukmuenster.de (M.M.); 3 Department of Nuclear Medicine, University Hospital Essen, 45147 Essen, Germany; 4 Department of Urology, University Hospital Muenster, 48149 Muenster, Germany; katrin.schlack@ukmuenster.de (K.S.); martin.boegemann@ukmuenster.de (M.B.)

**Keywords:** PSMA, PSMA-617, radiomics, radionuclide therapy, mCRPC

## Abstract

**Simple Summary:**

PSMA Therapy has recently become an additional therapeutic option in advanced prostate cancer. In the present study, the predictive and prognostic value of radiomic features from pretherapeutic PSMA PET-MRI are analyzed. Twenty-one patients with advanced prostate cancer underwent PSMA-therapy, including pretherapeutic PSMA PET-MRI. Radiomic features from PET- and MRI-sequences were extracted and processed to select the features differentiating responders and non-responders. Out of ten independent radiomic features differentiating between these two groups, the feature interquartile range from the T2 weighted images revealed the highest accuracy. PSA response and higher T2 interquartile range values might have impact on survival. This proof-of-concept study applies radiomic analysis to pretherapeutic PSMA PET-MRI before PSMA therapy, providing new parameters with potential predictive and prognostic value.

**Abstract:**

^177^Lutetium PSMA-617 (Lu-PSMA) therapy in patients with metastatic castration resistant prostate cancer (mCRPC) has gained visibility through the ongoing phase III trial. The data on prediction of therapy outcome and survival out of pretherapeutic imaging parameters is still sparse. In this study, the predictive and prognostic value of radiomic features from ^68^Ga-PSMA-11 PET-MRI are analyzed. In total, 21 patients with mCRPC underwent ^68^Ga-PSMA-11 PET-MRI before Lu-PSMA therapy. The PET-positive tumor volume was defined and transferred to whole-body T2-, T1- and contrast-enhanced T1-weighted MRI-sequences. The radiomic features from PET and MRI sequences were extracted by using a freely available software package. For selecting features that allow differentiation of biochemical response (PSA decrease > 50%), a stepwise dimension reduction was performed. Logistic regression models were fitted, and selected features were tested for their prognostic value (overall survival) in all patients. Eight patients achieved biochemical response after Lu-PSMA therapy. Ten independent radiomic features differentiated well between responders and non-responders. The logistic regression model, including the feature interquartile range from T2-weighted images, revealed the highest accuracy (AUC = 0.83) for the prediction of biochemical response after Lu-PSMA therapy. Within the final model, patients with a biochemical response (*p* = 0.003) and higher T2 interquartile range values in pre-therapeutic imaging (*p* = 0.038) survived significantly longer. This proof-of-concept study provides first evidence on a potential predictive and prognostic value of radiomic analysis of pretherapeutic ^68^Ga-PSMA-11 PET-MRI before Lu-PSMA therapy.

## 1. Introduction

Metastatic castration resistant prostate cancer (mCRPC) remains a challenge in clinical oncology despite promising results for new therapeutic agents [1]. Lutetium PSMA-617 (Lu-PSMA) therapy has gained visibility through the ongoing phase III trial in mCRPC patients. Previous prospective and retrospective analysis have already suggested a clinical efficacy and favorable tolerability [2,3].

In patients undergoing Lu-PSMA therapy, a PSA decline greater than 50% from baseline is achieved in 45–66% of them [3,4,5,6]. This threshold is used in many studies to define the response to therapy and is associated with survival [4,7,8,9]. The identification of non-responders is of the utmost importance to enable the early application of other palliative therapies within mCRPC patients. Predictive biomarkers that are associated with responses to Lu-PSMA therapy are needed.

Single parameters from morphologic or functional PET imaging are predictive and prognostic factors in prostate cancer patients: A high tumor volume from morphological imaging is a negative prognostic factor in prospective studies [10,11]. The quantitative volumetric PET parameter tumor volume (TV) and a total lesion quotient (TLQ) were excellent prognosticators of overall survival in prostate cancer patients undergoing PSMA therapy [5]. However, there are contradictory reports as by Ferdinandus et al. reporting no statistically significant correlation between PSMA-TV and overall survival in 50 patients treated with Lu-PSMA [12].

The data on radiomics/machine learning approaches for the assessment of predictive or prognostic value of PSMA-PET are still sparse [13,14]. However, these techniques are frequently applied to morphological imaging [15,16]. Many studies on MRI-based radiomics focus on the prediction of histopathological properties and prognostic information from pre-surgical work-up [17]. Radiomic data on post-surgical MRI and in biochemical recurrence are limited [18].

The first results on the extraction of PET-based textural features beyond standard uptake values (SUV) showed promising results for the correlation to PSA change during and after PSMA therapy [19]. First reports on the prediction of response to PSMA-therapy [20] and prediction of outcome after PSMA-therapy [21] with the help of radiomic features of pretherapeutic PSMA-PET-CT have shown emerging results.

This study aimed to analyse the role of conventional and radiomic features extracted from ^68^Ga-PSMA PET-MRI prior to PSMA therapy for the prediction of biochemical response and survival.

## 2. Materials and Methods

### 2.1. Patients

All patients that underwent ^68^Ga-PSMA-11 PET-MRI before radioligand therapy (RLT) were included in this retrospective study. The eligibility criteria for Lu-PSMA therapy followed institutional criteria and international guidelines and included the following: progressive disease in imaging, mCRPC, sustained androgen deprivation therapy, history of chemotherapy (if no contraindication was present at least one line of taxane chemotherapy), PSMA avid lesions in ^68^Ga-PSMA-11 PET-MRI, hematological reserve, as well as normal liver and renal function parameters [22].

Lu-PSMA therapy was performed on a case-by-case decision basis in the clinical routine and only after recommendation by the interdisciplinary tumor board. Informed consent for the therapy was obtained from all patients. All procedures performed in studies involving human participants were in accordance with the ethical standards of the institutional and/or national research committee and with the 1964 Helsinki declaration and its later amendments or comparable ethical standards. Data analysis was done retrospectively and was approved by the local ethics committee (No. 2016-585-f-S, Ethikkommission der Ärztekammer Westfalen-Lippe und der Westfälischen Wilhelms-Universität Münster).

### 2.2. ^68^Ga-PSMA-PET-MRI

All data were acquired on a combined 3-Tesla PET-MRI system (Biograph mMR, Siemens Healthineers, Erlangen, Germany). Patients were asked to void their bladder right before the start of the examination. Patients were positioned supine with arms next to the body. Head/neck and body surface coils were used for MR imaging.

Image acquisition was initiated 60 min after administration of ^68^Ga-PSMA-11. Before starting the PET acquisition, a two-point Dixon VIBE sequence for attenuation correction of PET data was acquired. Standard MR-whole body imaging protocol comprised a transversal T2 weighted half-Fourier acquisition single-shot turbo spin echo (HASTE) (TE/TR [ms]: 102/1500; FOV: 380 × 420; matrix: 218 × 320; slice thickness [mm]: 6), a coronal (TE/TR [ms]: 1.06/3.16; field of view (FOV): 400 × 450; matrix: 195 × 288; slice thickness [mm]: 3) and transversal 3D T1 volumetric interpolated breath hold examination (VIBE) sequence with fat suppression (TE/TR [ms]: 1.96/4.47; field of view (FOV): 341 × 420; matrix: 182 × 320; slice thickness [mm]: 3) before and after application of contrast agent (Gadovist^®^; Bayer Healthcare, Leverkusen, Germany). PET reconstruction was done using manufacture standard tools.

### 2.3. Lu-PSMA Therapy and Outcome

Median time from PET acquisition until Lu-PSMA therapy start was 26 days (range: 10–53 days). Lutetium was provided by ITG Isotopes Technology, Garching, Germany. The PSMA-617 precursor was provided by ABX (ABX GmbH, Radeberg, Germany). Syntheses of [177Lu]Lu-PSMA-617 (Lu-PSMA) followed previously described procedures [23]. Lu-PSMA was administered in a median 8-week interval (range: 6–9 weeks) with a median dose of 6.4 GBq. Following guidelines for PSMA-therapy and recently published data, therapy was discontinued in case of disease progression, severe adverse reactions or altered therapy regime [22]. During therapy, hematological, liver and renal function parameters and tumor marker PSA were regularly checked. Patients had regular blood sample checkups and imaging if clinically needed. Maximum change of PSA-value compared to baseline (first cycle) was assessed. A PSA decline of ≥ 50% was defined as biochemical response. Date of pretherapeutic PSMA-PET was used as a baseline for the calculation of survival parameters.

### 2.4. Image Analysis and Radiomic Feature Extraction

**Figure 1:**
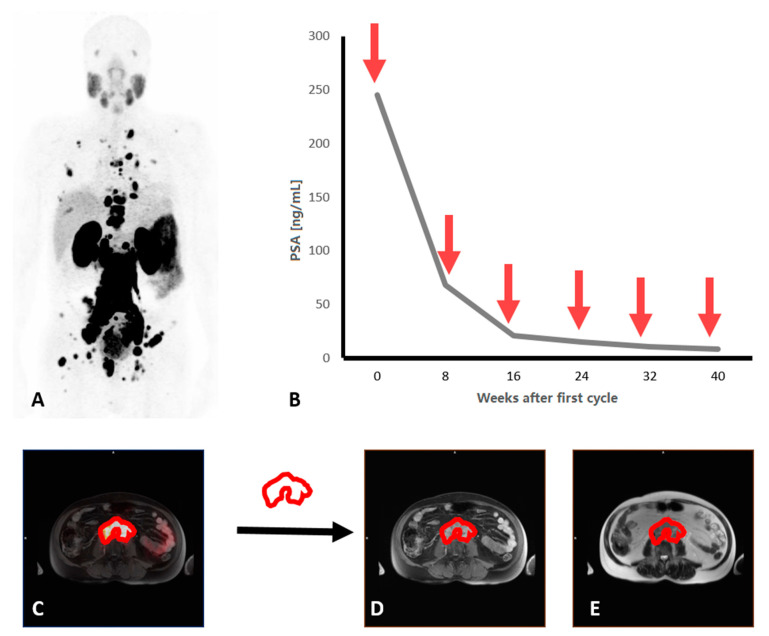
A heavily pretreated patient with metastatic castration resistant prostate cancer and high PSMA uptake in skeletal and lymph node metastases in maximum intensity projection of ^68^Ga-PSMA-11-PET-MRI (**A**). During six cycles of ^177^Lu-PSMA-617-therapy (red arrows), the tumor marker PSA decreased by 97% (**B**). PET positive volume was defined by application of a threshold and manual adjustment (**C**). This volume was transferred to MRI-sequences (**D**,**E**) to extract the radiomic features.

A threshold-based approach, using 3.0 SUV was used to define PSMA-PET-positive volume on the PET dataset [12,24]. Two experienced PET/MRI readers blinded for clinical data (one trained nuclear medicine physician and one trained radiologist) each independently adjusted lesion volumes manually and removed physiological uptake sites. Output variables were SUVmean, SUVmax, SUVmedian and total PSMA-PET-positive volume. For image segmentation, the reader-specific label map volume based on the PSMA-PET positive volume was then transferred to whole body T2-, T1- and contrast-enhanced T1-weighted MRI-sequences (Figure 1). Radiomic features from labelled PET and MRI-sequences were extracted twice, each by the same independent readers for inter-observer analysis, and included 162 first-order logic features and 216 gray level co-occurrence matrix (GLCM) features, as described elsewhere [25]. Image analysis and feature extraction was performed by using a freely available software package (3D slicer, version 4.11.2).

### 2.5. Radiomic Feature Selection and Dimension Reduction for Differentiation of PSA Response

Feature selection and dimension reduction were necessary, as the number of radiomic features (*n* = 378) exceeded the number of patients (*n* = 21) [26,27]. Inter-observer reproducibility of the textural features was assessed by calculating the Concordance Correlation Coefficient (CCC) for each of the features as a measure of the intra-class correlation. Features with a coefficient ranging from 0.8 to 1 were considered “excellent” and included in further analysis [28]. After feature normalization using z-score standardization, the dataset was randomly subdivided into a balanced training and test dataset (70/30 ratio). Further feature reduction was performed only on the training dataset using a Boruta machine learning algorithm. The Boruta algorithm applies a Random Forest algorithm by performing a top-down search for relevant features. Original attributes’ importance are compared with importance achievable at random, and irrelevant features are progressively eliminated to stabilize the model [29]. Subsequently, a correlation matrix was calculated since there is no relevant gain in information in closely correlated features, as described elsewhere [27,30]. Finally, for selecting features that allow differentiation of biochemical response (PSA decrease > 50%) logistic regression models were fitted. Diagnostic accuracy of the features was evaluated by receiver operating characteristic (ROC) calculating the area-under-the-curve (AUC). An optimal cut-off value was defined using Youden’s Index. Radiomic feature selection and dimension reduction was performed by using an open-source software package (R/R studio, version 4.0.5; R Foundation, Vienna, Austria).

### 2.6. Survival Analysis

Selected features of radiomic analysis were tested for their prognostic value (overall survival) in all patients with Kaplan–Meier analysis and log-rank test. Features with significantly different Kaplan–Meier survival curves were included into multivariate analysis. In addition, clinical parameter Eastern Cooperative Oncology Group (ECOG) status and Gleason score, blood-based biomarkers hemoglobin (Hb) and alkaline phosphatase (ALP) were included into multivariate analysis. Based on the review by Manafi-Farid et al., the Gleason score is confirmed to have positive impact on response prediction or on longer OS. Performance status (ECOG- score) and ALP plausibly have impact [31]. In a previous radiomic analysis, Hb had predictive value and was therefore added to this multivariate analysis [21]. 

### 2.7. Statistical Analysis

Demographic and clinical parameters were reported as total number and percentage, mean and standard deviation or median and range or 95% confidence interval (CI), as appropriate. *p*-values < 0.05 were considered to be statistically significant. Descriptive statistics and survival analysis were performed using the SPSS Statistics version 26 (SPSS Inc., Chicago, Illinois, USA).

## 3. Results

### 3.1. Patient Characteristics

Twenty-one patients (median age 69 years; range: 47–82) with mCRPC that underwent ^68^Ga-PSMA-PET-MRI before and after RLT with Lu-PSMA were included in this retrospective analysis. All patients previously received at least one taxane chemotherapy treatment (81%) or Abiraterone/Encalutamide (100%). Eight patients achieved biochemical response (PSA decline > 50%) in post-therapy follow-up. The median Hb was 10.5 g/dl (range: 8.1–15.6), and the median ALP was 187.0 U/I (range: 53.0–638.0). The median ECOG status was one (range 0–2). Detailed patient characteristics are presented in Table 1.

### 3.2. Radiomic Features and PSA response

**Figure 2:**
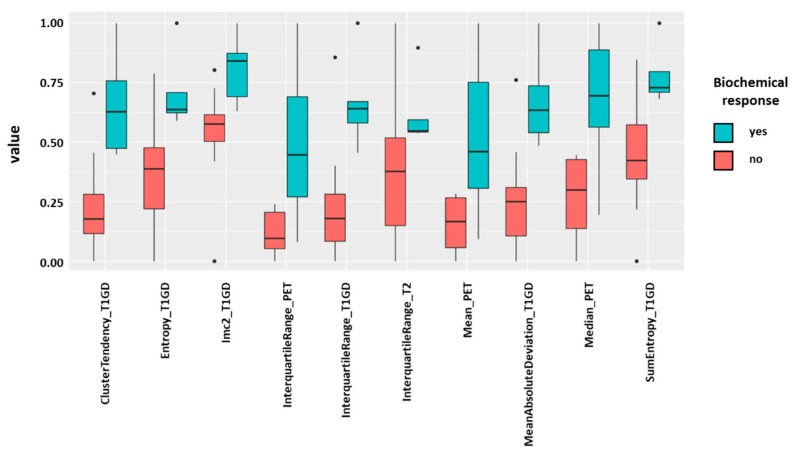
Ten independent features of pre-treatment ^68^Ga-PSMA-PET-MRI differentiated well between responders (green) and non-responders (red) to ^177^Lu-PSMA-617-therapy. Biochemical response was defined as any PSA decline above 50% from baseline.

**Figure 3:**
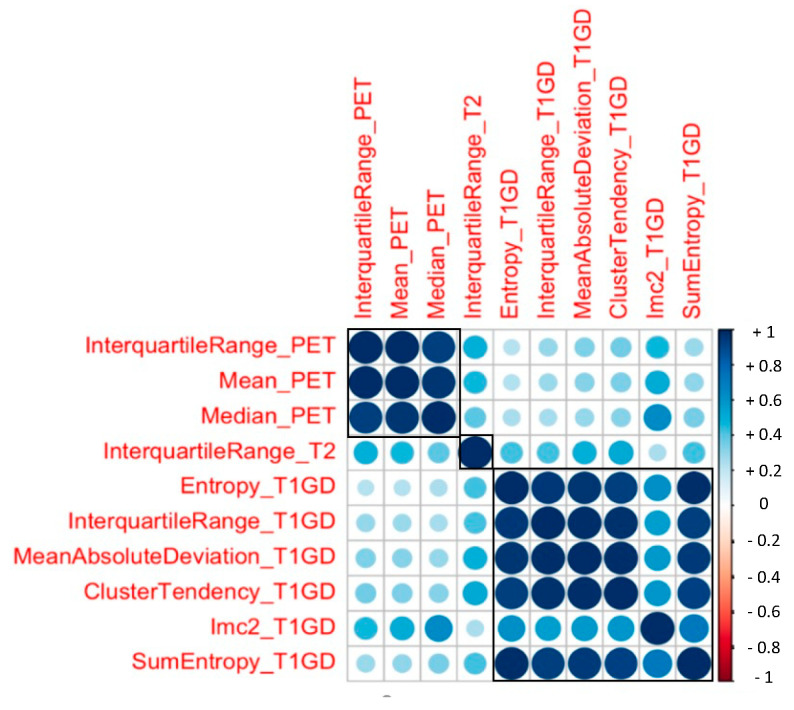
Correlogram including ten most important independent radiomic features. Three clusters of textural features became apparent (boxes). These indicate a strong correlation between parameters of the same imaging method. Blue circles indicate positive correlation, red circles negative correlation.

After the multistep dimension reduction, 10 independent features consisting of 3 PET-derived (interquartile range PET, mean PET, median PET), one T2-derived (interquartile range T2) and four T1-post-GD-derived parameters (interquartile range T1GD, Entropy T1 GD, mean absolute deviation T1GD, cluster tendency T1GD, Imc2 T1GD, SumEntropy T1GD) differentiated well between responders and non-responders to PSMA-therapy (Figure 2). Three clusters of radiomic features became apparent in the correlation matrix. However, the features from different modalities and sequences did not correlate significantly (Figure 3).

When assessing the predictive value of these tissue factors, only the ROC analysis for the discrimination of responders and non-responders to PSMA therapy (PSA decline > 50%) showed a good accuracy for the T2 interquartile range (AUC = 0.83). A threshold was defined and applied following Youden’s index resulting in a binary code for T2 interquartile range.

### 3.3. Survival Analysis

**Figure 4:**
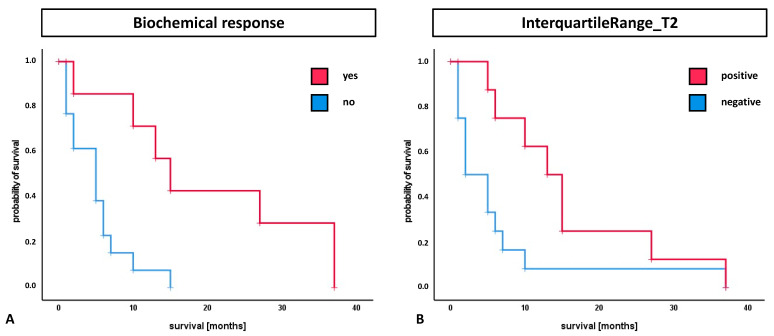
Survival analysis for the parameters of biochemical response (PSA decline > 50%) and InterquartileRange_T2. (**A**) The median OS in patients with biochemical response was significantly higher compared to patients with different PSA change (15.0 vs. 5.0 months; *p* = 0.003). (**B**) Patients with a positively binary-coded T2 interquartile range showed significantly longer median OS compared to patients with a negative value (13.0 vs. 2.0 months; *p* = 0.038).

The median overall survival (OS) was 6.0 months. The median OS in patients with PSA decline greater than 50% (biochemical response definition) was significantly higher compared to patients with less than 50% decrease or increasing PSA (15.0 vs. 5.0 months; *p* = 0.003) (Figure 4). Patients with a positive binary-coded T2 interquartile range showed significantly longer median OS compared to patients with a negative value (13.0 vs. 2.0 months; *p* = 0.038) (Figure 4). The multivariate analysis included previously established clinical parameters (Gleason Score, ECOG status, ALP, Hb), biochemical response and radiomic features of the T2 interquartile range with impact on survival in the Kaplan–Meier analysis. Here, the positively binary-coded T2 interquartile range and the biochemical response were found to be independent parameters with significant influence on survival (*p* = 0.023 and *p* = 0.028).

## 4. Discussion

MCRPC remains a challenge in clinical oncology, although the number of new therapeutic agents is steadily increasing [1]. Lu-PSMA therapy, as one of these new agents, has gained visibility through an ongoing phase III trial with positive endpoint results in these patients. Previously, the prospective and retrospective analysis with different patient collectives have shown favorable outcomes and low toxicity [2,3].

However, in about 33–55% of Lu-PSMA therapy patients, a biochemical response cannot be achieved [3,4,6]. Thus, it is of the utmost importance to identify biomarkers that are associated with response to Lu-PSMA therapy and outcomes. This would optimize patient selection for Lu-PSMA therapy and enable early changes in patient management.

Prior to therapy, PSMA-PET is the gatekeeper for the evaluation of the uptake and extent of disease. However, using different quantitative PET uptake parameters, most studies were not able to predict response to therapy. Thus, the decision for therapy cannot be based on single uptake parameters, such as SUVmax in certain lesions [31,32]. Computer-aided diagnostics of radiomic features might overcome these limitations of measuring single parameters or subjective visual analysis [33]. Currently, radiomic approaches are more frequently applied to morphological CT or MRI than to PET datasets [15]. The first reports on PSMA-PET [13] and PSMA-PET-MRI [34] focus on prostate cancer patients at an earlier stage of the disease. The data on radiomic analysis of pretherapeutic PSMA-PET in patients undergoing Lu-PSMA therapy are still sparse.

Out of 378 radiomic features, both from PET and MRI, we determined the most relevant in a multistep dimension reduction approach for the analysis of PSA response after Lu-PSMA therapy. Ten independent radiomic features differentiated well between responders and non-responders. We identified the interquartile range from the T2-weighted images as the feature with the highest predictive accuracy on PSA response. Moreover, the T2 interquartile range was found to be an independent parameter with a significant influence on survival. In line with the findings from other studies, conventional PET parameters, such as SUVmax or SUVmean, did not have predictive value in this study [8,31,32]. Especially in advanced cancer stages, tumor heterogeneity might not be reflected by conventional imaging parameters [35]. Radiomic analysis of hybrid imaging, including functional and morphological parameters, might overcome these limitations.

In a comparable study design, Moazemi et al. performed a radiomic analysis on PET/CT data for the prediction of treatment response [20] and for the prognostication of overall survival [21] in patients treated with Lu-PSMA. They analyzed 80 radiomic features both from PET and CT in a cohort of *n* = 72 and 73 features in *n* = 83 patients with advanced prostate cancer, respectively. In contrast to our study, they performed data augmentation by using the least absolute shrinkage and selection operator (LASSO) regularization method to select the most relevant features. However, they were also able to establish a radiomic signature (e.g., size variation, kurtosis) with predictive and/or prognostic power [20,21].

Although these data confirm the potential of radiomic and textural analysis in the work-up of patients undergoing Lu-PSMA therapy, novel models should also include clinical parameters. Moreover, when creating a module for the prediction of response to therapy or outcome, the underlying pathology should be adequately characterized. The Gleason score is therefore one of the parameters predicting the response to Lu-PSMA therapy, as it reflects tumor aggressiveness [31]. However, in the present study, the Gleason score did not show a prognostic impact, probably because of a very high Gleason score in the majority of patients (median nine). Similarly, performance status was comparably high in these patients (median one) not resulting in a prognostic impact.

Combining these imaging-derived textural features with clinical parameters might allow for more powerful prediction models. Thus, previous studies could show that not only imaging derived features, but also blood based parameters, such as Hemoglobin, had predictive and prognostic value in the underlying patient cohort [7,12,21,32]. However, the predictive and prognostic value of blood-based monoanalyte markers ranges from plausible to controversial [31]. In line, in this study, blood-based parameters Hb and ALP did not have prognostic value. In the future, the prediction of therapy responses and prediction of survival will be supported by multianalyte markers as circulating tumor RNA or DNA, as well as for radioligand therapies [36,37].

This study is limited by its retrospective study design and low number of patients, including a potential selection bias. This small number of patients especially limits the survival analysis. The segmentation of tumoral lesions is always a matter of discussion. In this study, segmentation was performed based on the PET dataset with a fixed threshold with the manual elimination of physiological uptake, as previously published [12]. New artificial intelligence applications already allow for automatic whole body tumor assessment, including the automatic elimination of physiological uptake not only for FDG-PET [38] but also for PSMA-PET/CT [39,40]. Although performing a multistep feature dimension reduction approach was only performed in the training dataset to ensure the generalizability of the statistical model, the number of patients in the test dataset is relatively small. Moreover, we only analyzed data from one institution acquired from one PET/MRI scanner. Since some of the radiomic features were found to be dependent on acquisition parameters such as type of PET, CT or MRI system, the reconstruction kernel and the contrast media, the results of this radiomic analysis might need to be adapted for future use with other systems [30,41]. Furthermore, the results need to be validated in a prospective study with a larger number of cases. PET-MRI might offer additional imaging parameter for response assessment and prediction after Lu-PSMA therapy. Prospective evaluation of sophisticated algorithms [34] with correlation to histopathology [13] and/or liquid biopsy might pave the way for clinical use of radiomics and learning applications.

## 5. Conclusions

A radiomics analysis of pretherapeutic ^68^Ga-PSMA-11 PET-MRI prior to ^177^Lu-PSMA-617 therapy may potentially offer predictive and prognostic parameters. Beyond imaging-based parameters, blood-based biomarkers and previous treatments should be included in future studies.

## Figures and Tables

**Table 1:** Patient characteristics. Values are presented as median (interquartile range) or frequency (percentage of all patients).

Patient Characteristics	Total Cohort	
**Age (years)**	69	(range: 47–82)
**Gleason Score**	9	(range: 6–10)
**Metastases location**		
Bone	20	[95.2]
Lymph nodes	18	[85.7]
Liver	8	[38.1]
Lung	3	[14.3]
**Previous therapies**		
Abiraterone	18	[85.7]
Enzalutamide	17	[81.0]
Docetaxel	17	[81.0]
Cabazitaxel	8	[38.1]
**PSMA-therapy**		
Prostate-specific antigen at first cycle (ng/ml)	217.8	[2.6–3294]
Number of cycles	3	(range: 1–8)
Administered activity per cycle (GBq)	6.2	(range: 5.9–7.5)
Cumulated activity (GBq)	17.6	(range: 6.0–49.7)

## Data Availability

Analyzed data are stored at the Department of Nuclear medicine, University hospital Münster, Germany, and are available on reasonable request if not already included in this article.
